# Effects of Probiotic (*Bacillus subtilis* DE111) Supplementation on Immune Function, Hormonal Status, and Physical Performance in Division I Baseball Players

**DOI:** 10.3390/sports6030070

**Published:** 2018-07-26

**Authors:** Jeremy R. Townsend, David Bender, William C. Vantrease, Philip A. Sapp, Ann M. Toy, Clint A. Woods, Kent D. Johnson

**Affiliations:** Exercise and Nutrition Science Graduate Program, Lipscomb University, Nashville, TN 37204, USA; wdbender1@mail.lipscomb.edu (D.B.); vantreaswc@mail.lipscomb.edu (W.C.V.); psapp@mail.lipscomb.edu (P.A.S.); ann.toy@lipscomb.edu (A.M.T.); clint.woods@lipscomb.edu (C.A.W.); kent.johnson@lipscomb.edu (K.D.J.)

**Keywords:** gut microbiota, resistance training, cytokines, athletic performance, sport nutrition

## Abstract

We sought to determine the effects of probiotic supplementation (*Bacillus subtilis* DE111; 1 billion CFU∙d^−1^) on markers of immune and hormonal status in collegiate male athletes following 12 weeks of offseason training. Twenty-five Division I male baseball athletes (20.1 ± 1.5 years, 85.5 ± 10.5 kg, 184.7 ± 6.3 cm) participated in this double blind, placebo-controlled, randomized study. Participants were randomly assigned to a probiotic (PRO; *n* = 13) or placebo (PL; *n* = 12) group. Pre- and post-training, all athletes provided resting blood and saliva samples. Circulating concentrations of testosterone, cortisol, TNF-α, IL-10, and zonulin were examined in the blood, while salivary immunoglobulin A (SIgA) and SIgM were assayed as indicators of mucosal immunity. Separate analyses of covariance (ANCOVA) were performed on all measures collected post intervention. No differences in measures of body composition or physical performance were seen between groups. TNF-α concentrations were significantly (*p* = 0.024) lower in PRO compared to PL, while there were no significant group differences in any other biochemical markers examined. A main effect for time was observed (*p* < 0.05) for increased testosterone (*p* = 0.045), IL-10 (*p* = 0.048), SIgA rate (*p* = 0.031), and SIgM rate (*p* = 0.002) following offseason training. These data indicate that probiotic supplementation had no effect on body composition, performance, hormonal status, or gut permeability, while it may attenuate circulating TNF-α in athletes.

## 1. Introduction

Athletes regularly engage in rigorous exercise training, which leads to accumulating amounts of physical stress. While daily moderate intensity physical activity has been shown to have positive effects on the immune system [1], prolonged periods of intense training and competition may lead to immune dysregulation [2,3,4]. As a result of mucosal and systemic immune suppression, it is common for competitive athletes to become susceptible to infections, which may reduce the frequency and quality of physical training and athletic competition [5]. In addition to being vulnerable to infection, overly fatigued athletes are found to have altered levels of pro and anti-inflammatory cytokines in circulation [6,7]. For instance, elevated circulating TNF-α in elite male rowers was significantly associated with depressed mood, sleep disturbances, and physical stress [8]. Moreover, TNF-α acts to impair protein synthesis in skeletal muscle by decreasing messenger RNA (mRNA) translational efficiency [9]. This combination of factors may limit an athlete’s ability to properly recover from acute training bouts and may ultimately impair training adaptations.

While athletes are often subjected to excessive levels of physical stress as a byproduct of training demands, other stressors are often overlooked. Specifically, collegiate athletes regularly engage in periods of high physical stress accompanied with prolonged travel, academic rigor, and other physiological stressors. College athletes who are under a large amount of physical and academic stress have recently been shown to be more susceptible to sustaining injury during these times of increased strain [10]. To counter this, biomarker monitoring is gaining momentum in the athletic realm as a method to detect periods of excessive negative physiological stress [11]. Furthermore, it has been suggested that utilizing an assembly of diverse biomarkers may provide the most effective strategy in evaluating intricate balance of anabolic and catabolic processes in athletes [11,12].

To attenuate the increasing levels of physiological strain associated with training, athletes often implement nutritional strategies to support immune health. Probiotic supplementation, for instance, is a strategy which is receiving considerable attention as a countermeasure for training-induced stressors [13]. Probiotics are live organisms that when consumed, impose a wide array of beneficial physiological effects on humans, most notably promoting improved gut microbiota [14]. These microorganisms have been shown to exert immunomodulatory effects [15] by decreasing pro-inflammatory cytokines in circulation [16] and supporting mucosal defense [17,18]. In athletes, probiotics have been reported to reduce the number, duration, and severity of infections [18,19,20]. Thus by improving resistance to infection, attenuating low-grade inflammation, and improving nutrient absorption, probiotic supplementation may be a practical strategy to support athlete health and adaptation [13,21]. However, as the effects of probiotics are strain-specific and not all studies have observed a positive effect of supplementation on immune function and sickness [22,23], each strain must be investigated independently to elucidate its unique effects.

While probiotics appear to have a generally positive effect on athlete immune function, studies regarding its efficacy on improving exercise performance are less clear. In endurance athletes, a multi-strain probiotic significantly improved time until fatigue in males running at 80% of their ventilatory threshold [24] whereas others have reported no effect of probiotics on performance [17,19,20]. Regarding resistance exercise, Jager et al. [25] found that co-ingestion of protein with *Bacillus coagulans* GBI-30, 6086 attenuated range of motion decrements in recovery following an intense bout of resistance exercise possibly by improving nutrient absorption [26]. Furthermore, 10 weeks of *Bacillus subtilis* DE111 supplementation in conjunction with adequate post-workout nutrition was shown to improve body composition in female collegiate athletes while exerting no effect on physical performance [27]. Despite recent interest, only a limited amount of investigations have investigated the effect of probiotic supplementation on training outcomes in resistance-trained individuals [27,28,29] and more data is needed to delineate their effects on performance.

Therefore, the aim of the present study was two-fold. First, we sought to examine the effects of 12 weeks of daily probiotic supplementation on immune and hormonal profiles in college athletes during a period of increased academic and physical stress. Second, we evaluated the effect daily probiotic supplementation on physical and performance adaptations in Division I collegiate baseball players following 12 weeks of offseason training. With this investigation, we sought to address gaps in the literature regarding probiotic supplementation in team sport athletes, as well as further explore potential mechanism by which probiotics may improve athlete health and performance.

## 2. Materials and Methods

Twenty-five Division I male baseball athletes (20.1 ± 1.5 years, 85.5 ± 10.5 kg, 184.7 ± 6.3 cm) participated in this double blind, placebo-controlled, randomized study. Participants were randomly assigned to a probiotic (PRO; *n* = 13) or placebo (PL; *n* = 12) group, utilizing blocked counterbalanced randomization based on participant order in reporting to the lab to ensure groups were as equal in n-size as possible. Following an explanation of all procedures, risks, and benefits, each participant provided their written informed consent prior to participation in this study. The research protocol was approved by the Institutional Review Board of the University prior to participant enrollment. Exclusion criteria included the use of probiotic supplementation, ergogenic aids, or suffering from any medical, muscular, or metabolic contraindications. 

### 2.1. Study Protocol

Participants reported to the Human Performance Lab (HPL) on two separate occasions at the beginning (PRE) and end (POST) of the 12-week training intervention following a 10-h overnight fast. Additionally, athletes were instructed to report to the lab hydrated while abstaining from caffeine, alcohol, and vigorous exercise for at least 24 h prior to both laboratory testing sessions. During these visits the participants were tested for body composition, muscle thickness, and provided biological samples. Furthermore, athletes reported to their strength and conditioning coordinator on two separate occasions pre- and post-training, to measure 1RM for squat and deadlift along with testing pro-agility, 10-yard sprint, and standing long jump. Pre-training, all 1RM sessions began at the beginning of the fall semester during the first week of classes. Post-training, 1RM and performance testing occurred the week prior to final examinations. As one of our aims was to investigate biomarkers of fatigue and immune function during a stressful period, we chose to conduct our post-training biochemical sample collection during final examination week [10]. Additionally, as winter months have been shown to produce additional challenges to the immune system [20], our post-testing biochemical sampling occurred in a winter month as well (December). 

### 2.2. Body Composition

#### 2.2.1. Air Displacement Plethysmography

Body density was estimated using air displacement plethysmography using the BODPOD^®^ (COSMED, Rome, Italy). Prior to each test, the BODPOD was calibrated according to the manufacturer’s instructions using a two-point calibration. Prior to testing, athletes were instructed to wear tight fitting compression shorts and a swimming cap, as well as to remove all metal, including jewelry and watches. Body mass was measured to the nearest 0.01 kg using the system’s calibrated scale. All athletes were instructed to sit in the chamber, breath normally, and to minimize any movement. A minimum of two trials were performed. If measurements were not within 150 mL of each other, a third trial was conducted. Thoracic gas volume was estimated using the BODPOD software, which uses standard prediction equations and has demonstrated no difference compared to measured lung volumes [30].

#### 2.2.2. Bioelectrical Impedance Analysis

Total body water (TBW) was determined using multi-frequency bioelectrical impedance analysis (BIA) using the InBody^®^ 570 Body Composition Analyzer device (Biospace, Inc., Seoul, Korea). Body composition from BIA is obtained from the measures of resistance and reactance when an electrical current travels throughout the body. Prior to each assessment the participants’ hands and feet were thoroughly cleaned with InBody^®^ provided tissues. Age, height, and sex were manually entered, while a scale positioned within the device assessed body mass. The participant was then instructed from the software to stand fully erect on the measurement electrodes situated on the platform and to hold hand electrodes, with arms extended, without touching the sides of their body. Participants were asked to refrain from moving or talking until the assessment was completed. It has previously been shown that BIA is a valid measurement tool for determining TBW when compared to a deuterium oxide technique [31].

#### 2.2.3. Three-Compartment Model (3C-W)

The criterion percent body fat (%BF) was estimated using the three compartment-water (3C-W) model described by Siri [32]. The equation includes measurements of body density (from the BODPOD), TBW (from the BIA), and body mass (BM). The equation for %BF is listed below:

%BF = ((2.118/Body density) − (0.78 × TBW(L)/BM(kg)) − 1.354) × 100

### 2.3. Muscle Ultrasonography

Non-invasive measurements of muscle thickness (MT) were collected using B-mode ultrasound imaging with a 12 MHz linear probe (General Electric LOGIQ P5, Wauwatosa, WI, USA). Measurements for the rectus femoris (RF) were taken at 50% of the distance from the anterior, inferior suprailliac spine to the most proximal point of the patella [33]. Vastus lateralis (VL) measurements were taken in the same fashion as previously stated; however, the sampling location was determined by 50% of the straight-line distance between the greater trochanter and the lateral epicondyle of the femur [34]. Prior to image collection, participants laid supine for 5 min and the probe was coated with a water-based conduction gel. For measurements of MT, the probe was oriented longitudinally in the sagittal plane parallel to the muscle tissue without depressing the skin. Once images were collected, analysis was completed using ImageJ software (version 1.45s; National Institutes of Health, Bethesda, MD, USA). MT was determined from the still image as the distance between the inferior border of the superficial aponeurosis and the superior border of the deep aponeurosis. Intraclass correlation coefficients (ICC_3,k_), standard error of measurements (SEM), and minimal differences (MD) for the ultrasound technician were calculated for the RF MT (ICC_3,k_ = 0.99, SEM_3,k_ = 0.07, MD = 0.19 cm) and VL MT (ICC_3,k_ = 0.99, SEM_3,k_ = 0.01, MD = 0.03 cm) from analysis of 10 individuals separated by 24 h.

### 2.4. Dynamic Strength Testing

One-repetition maximum (1RM) strength was assessed in squat and dead lift exercises. All 1RM testing was performed using methods previously described [35]. Prior to testing, each athlete completed a general warm-up led by the strength and conditioning coach, which included jogging and a dynamic warm-up. Each athlete performed two warm-up sets using a resistance of approximately 40–60% and 60–80% of her perceived maximum, respectively. For each exercise, 3–4 subsequent trials were performed to determine the 1RM. A 3–5 min rest period was provided between each trial. Trials not meeting the range of motion criteria for each exercise or where proper technique was compromised were discarded. 

### 2.5. Performance Testing

#### 2.5.1. Ten-Yard Sprint

The athletes then completed a standardized general and dynamic warm-up that was consistent with their normal training habits and led by the team’s strength and conditioning coach. A pair of cones and tape affixed to the floor were positioned to denote the “starting line”. The athletes were instructed to take their preferred starting stance at the starting line and to begin each maximal trial at their ready. Sprint times were quantified with infrared timing gates with 0.01 s precision (Brower Timing Systems, UT, USA). The best of three trials was recorded and used for analysis.

#### 2.5.2. Pro-Agility Test

For the pro-agility test, three cones were placed parallel, five meters apart. The athletes set up for the test in a straddle position facing the middle cone. On their ready, the athletes were instructed to pivot to their right and accelerate as quickly as possible to a cone 5 m away and then upon touching the first cone, pivot again to their left and sprint the 10 m distance to the furthest cone. Upon touching this cone, the athletes once again pivoted to the right to return to the middle cone as quickly as possible. During each change in direction, the athletes were asked to touch the ground next to the cone. Trials where the athlete failed to touch the ground were discarded. Time for the pro-agility was captured with laser timing gates with 0.01 s precision (Epic Combine Timer, Lincoln, NE, USA). Athletes were allowed three attempts and the fastest time measured in seconds was recorded. 

#### 2.5.3. Standing Long Jump

Standing long jump performance was assessed using a pre-marked (±0.5 in) commercial mat (Sportime, LLC, Norcross, GA, USA). Prior to the test, each athlete stood with both feet placed in the marked “starting area” on the mat. Athletes were instructed to perform a maximal horizontal long jump. Standing long jump distance was determined by furthest distance reached following three maximal countermovement jump attempts performed from a standing position with feet shoulder width apart.

### 2.6. Supplementation Protocol

Both the PRO and PL groups completed daily supplementation for 12 weeks. The PRO supplement consisted of one billion colony forming units (CFU) *Bacillis subtilis* DE111 (Deerland Enzymes, Kennesaw, GA, USA). Following production, probiotic count was confirmed (1.2 billion CFU/capsule) via plate count method by the manufacturer at the beginning of the investigation. The placebo capsule consisted of maltodextrin, and athletes consumed their respective treatment (PRO or PL) in the form of a capsule, both treatments being identical in appearance. On training days, capsules were consumed immediately post-workout with a protein (whey protein isolate) and carbohydrate (dextrose) recovery drink (27 g protein, 36 g carbohydrates, 2 g fat) in the presence of a study investigator. All athletes consumed the post-workout drink with their respective treatment capsule. Prior to each workout, a study investigator prepared the post-workout recovery drinks, organized the study product for consumption, and monitored compliance as supplementation occurred. On weekend or non-training days, athletes were provided their respective capsules in individual bags and were required to consume their supplement with a normal meal and return the used supplement bags. Capsules were kept in a dry cool area in the laboratory protected from light and moisture, while the athletes were instructed to store their supplement similarly on weekends. Throughout the entire 12-week intervention and the following biochemical and statistical analysis, all study investigators and participants were blinded as to which product each participant consumed. Following all data collection and analysis, study investigators were unblinded by the manufacturer providing the allocation codes, and the investigators became aware of the intervention.

### 2.7. Nutritional Analysis

During the training and supplement intervention, participants were asked to complete a three-day food log (two weekdays, one weekend day) on weeks one, nine, and 12. Dietary recalls were used to provide an estimate of total kilocalorie intake (kcal) and macronutrient distributions (carbohydrate, protein, and fat) of the athlete’s typical weekly diet. All dietary analysis was completed using the MyFitnessPal application (Under Armour Inc., Baltimore, MA, USA), which contains a large, detailed US-branded food database.

### 2.8. Saliva Sampling

Saliva and blood samples were obtained at two time points throughout the study (PRE, POST). All biochemical samples at POST were taken at the same time of day as PRE to avoid potential confounding influence of diurnal variations. Prior to saliva sampling, all athletes rested in a seated position for 5 min. With an initial swallow to empty the mouth, unstimulated whole saliva was collected by expectoration into a pre-weighed vial with eyes open, head tilted slightly forward, and making minimal orofacial movement. Study personnel then documented the saliva collection duration and weight of the sample. Saliva flow rate (mL/min) was determined by weighing with saliva density assumed to be 1.0 g/mL [36]. After collection, the sample tube was centrifuged at 3000× *g* for 15 min to remove cellular debris, which can negatively impact the accuracy of analysis [37]. The supernatant was then aliquoted and stored frozen at −80 °C for later analysis.

### 2.9. Blood Sampling

These blood samples were obtained using a single-use disposable needle with the athlete in a supine position for at least 15 min before sampling. All blood samples were collected into two Vacutainer^®^ tubes, one containing no anticlotting agent (6 mL) and the second containing K_2_EDTA (6 mL). The blood in the first tube was centrifuged immediately at 3000× *g* for 15 min while the second tube was allowed to clot at room temperature for 30 min and subsequently centrifuged at 3000× *g* for 15 min. The resulting plasma and serum were placed into separately labeled microcentrifuge tubes and frozen at −80 °C for later analysis.

### 2.10. Biochemical Analyses

Duplicate saliva samples were analyzed for secretory SIgA and SIgM concentrations using enzyme-linked immunosorbent assay (ELISA) kits (SIgA: Salimetrics, State College, PA, USA; SIgM: Abcam, Toronto, ON, Canada). The intra-assay coefficient of variation for saliva SIgA was 3.31% and 7.54% for SIgM. The SIgA and SIgM secretion rate was then calculated by multiplying the concentration by the saliva flow rate.

Circulating plasma concentrations of TNF-α and serum concentrations of IL-10, zonulin, testosterone, and cortisol were assayed via commercially available ELSIA kits (ALPCO, Salem, NH, USA). To limit interassay variability, all samples for a particular assay were thawed once, and analyzed by the same technician using a FLUOstar Omega spectrophotometer (BMGLabtech, Ortenberg, Germany). All samples were analyzed in duplicate with a mean coefficient of variation of 4.05% for TNF-α, 7.45% for IL-10, 4.10% for zonulin, 4.89% for testosterone, and 3.48% for cortisol.

### 2.11. Offseason Training Protocol

All athletes completed the same triphasic undulating periodized resistance training program for 12 weeks (2–3 days week^−1^) (Table 1). Triphasic training is a common periodized resistance training program designed to allow an athlete to eccentrically and isometrically absorb energy before applying it in a dynamic movement [38]. This program consisted of three mesocyles (3–4 weeks) in which athletes emphasize a particular phase of movement (eccentric, isometric, concentric) while performing their core lifts. In addition to strength training, the athletes participated in team conditioning, agility, jumping, and sprint work (2–3 sessions week^−1^). These workouts consisted of approximately 30–40 min of sport-specific skill development and conditioning-related work. All training sessions were performed under the supervision of a certified strength and conditioning specialist, as well as a certified athletic trainer.

### 2.12. Statistical Analysis

Prior to hypothesis testing, the Shapiro-Wilk test was used to evaluate the assumption of normality for dependent variables. Non-normally distributed data were transformed using the natural log (LN). To identify differences between the experimental conditions on changes in markers of immune and hormonal status, athletic performance, and body composition, an ANCOVA was performed on all measures collected at POST. Associated values collected at PRE were used as the covariate to eliminate the possible influence of initial score variances on the outcomes. Following any significant F-ratio, a paired-samples t-test was used to determine if significant difference existed between measures collected prior to and immediately following 12 weeks of training. Group differences were further assessed via effect sizes (ƞ^2^p; partial eta squared). Effect sizes were interpreted as small (0.01–0.059), medium (0.06–0.139), or large (>0.14) as previously recommended [39]. An alpha level was set at *p* ≤ 0.05, and all analyses were performed using SPSS version 24.0 (SPSS, Inc., Chicago, IL, USA).

## 3. Results

No significant differences were observed between groups for compliance, with all athletes achieving ≥92% with an average compliance of 98.8% across groups. No significant differences (*p* = 0.571) in average daily caloric intake were observed between PRO (2404 ± 494.3 kcals) and PL (2369 ± 616.3 kcals) groups. In addition, no significant differences were seen between groups in carbohydrate (*p* = 0.515; PRO: 262.2 ± 52.3 g vs. PL: 251.4 ± 62.6 g), protein (*p* = 0.871; PRO: 122.3 ± 33.3 g vs. PL: 128.0 ± 40.1 g), and fat (*p* = 0.274; PRO: 91.3 ± 28.7 g vs. PL: 86.5 ± 24.1 g) intakes. Furthermore, both PRO and PL supplements were well tolerated, and no adverse side effects were reported.

### 3.1. Strength, Performance, and Body Composition

Changes in strength, performance and body composition are presented in Table 2. There were no group differences observed between PRO and PL for any measure of strength, performance or body composition. Collectively, significant improvements (*p* < 0.001) were observed in squat 1RM, deadlift 1RM, pro-agility, and standing long jump as a result of 12 weeks of offseason training, while no improvement (*p* = 0.312) in 10-yard sprint time was found. Additionally, both groups experienced significantly increased (*p* < 0.001) RF and VL muscle thickness following training, while no improvements were seen for body fat % (*p* = 0.332). 

### 3.2. Biochemical Markers

Changes in biochemical markers are presented in Table 3. TNF-α concentrations were significantly (F = 5.859, *p* = 0.024, ƞ^2^ = 0.020) lower in PRO (∆: −0.25 ± 1.10 pg/mL, *p* = 0.453) compared to PL (∆: +0.36 pg/mL, *p* = 0.160). There were no other significant group differences in any other biochemical markers examined. However, a large (ƞ^2^ = 0.134) but statistically insignificant (*p* = 0.078) effect for lower cortisol concentrations in PRO (∆: −76.9 ± 222.1 nmol/L, *p* = 0.235) compared to PL (∆: +39.6 ± 126.03 nmol/L, *p* = 0.300) was observed at POST. Collectively, significant increases were observed for testosterone (*p* = 0.045), LN IL-10 (*p* = 0.048), SIgA rate (*p* = 0.031), and LN SIgM rate (*p* = 0.002) following 12 weeks of offseason training across groups. No main effects for time were observed in any other biochemical marker.

## 4. Discussion

The objective of this study was to examine the effect of daily probiotic supplementation on strength, performance, body composition and biochemical markers in Division I male college athletes. The results of this study indicate that probiotic supplementation did not provide any additional benefits on strength, performance, and body composition following offseason training compared to PL. Furthermore, it appears that probiotic supplementation appears to promote lower circulating TNF-α in resistance trained males. 

As probiotics have previously been shown to modulate pro- and anti-inflammatory cytokines in the body, it has been suggested that probiotics may support an athlete’s general immune health [13]. Additionally, intense physical training may cause damage to an athlete’s gut barrier, resulting in endotoxin translocation, oxidative stress, and a low-grade pro-inflammatory cytokine response [16,40,41,42]. In the present study, we found that 12 weeks of probiotic supplementation attenuated increases in TNF-α which were observed in the placebo group. Similar to our findings, probiotic supplementation reduced circulating TNF-α concentrations in endurance-trained men [16] while West et al. [20] found that probiotic supplementation likely decreased the magnitude of TNF-α concentrations following acute VO_2_max testing. TNF-α is a potent pro-inflammatory cytokine which is designed to serve an essential role in skeletal muscle remodeling [43,44]. However, pronounced levels of TNF-α have been linked suppressed protein synthesis, disordered sleep, and impaired muscular performance [8,9,45]. In this study, athletes in the placebo group experienced elevated TNF-α concentrations (2.78 ± 0.95 pg/mL) which were considerably lower than a previous study in humans which observed a significant relationship between increased TNF-α (8.09–8.78 pg/mL) and disordered sleep [8]. Moreover, while elevated levels of inflammation are generally regarded as detrimental to recovery and adaptation, our athletes experienced similar physical adaptations regardless of treatment group. It is also important to note that we studied athletes who completed a rigorous offseason training program, were in preparation for examination week, and were entering into the winter months. Thus, we cannot speak to the exact cause of the elevations in circulating TNF-α observed in the placebo group. Taken together, it is difficult to gauge the practical benefit of suppressed inflammation based on the measures included in our study. Nevertheless, during a time period where multiple stressors were present, it appears that probiotic supplementation may alter cytokine production in male collegiate athletes. 

While IL-10 concentrations in our study did not differ between groups, significant elevations were seen as a result of the offseason training program. Ibrahim et al. [29] found a significant increase in IL-10 concentrations following 12 weeks of circuit training alone and probiotic supplementation alone, while the combination of circuit training and probiotics trended towards a significant elevation post intervention. IL-10 is an anti-inflammatory cytokine that is generally elevated at post-resistance exercise as a means to suppress inflammation and begin the adaptation process [43,44]. While our participants refrained from strenuous activity prior to reporting to the lab, it is possible the observed modest elevation in IL-10 was a lingering anti-inflammatory response from their previous workout session. Future investigations utilizing additional biochemical sampling time points may provide context to interpret these findings.

Immunoglobulins are a heterogeneous group of antimicrobial proteins that appear as the immune system’s first line of defense in response to an antigen [46]. SIgA is the principal immunoglobulin involved in host defense and has been shown to be suppressed following intense acute [3,47,48] and chronic training [4,18]. Contrary to previous reports in endurance athletes [18] and military cadets [49], we found no effect of probiotics on indicators of mucosal immunity in our athletes. However, this is consistent with a number of studies in endurance athletes that found no differences in SIgA or SIgM between groups following probiotic interventions ranging from 4–12 weeks [19,20,50]. Additionally, another study found no differences in SIgA protein concentration or secretion rate in 24 male and 6 female professional athletes of various sports [17]. It is possible that due to the prolonged repetitive nature of endurance exercise, these athletes experience a larger volume of training-induced stress and are thus more prone to immune suppression than strength and team sport athletes. Team sport athletes likely spend a larger amount of offseason time indoors engaging in resistance training compared to endurance athletes (e.g., runners), who are constantly exposed to the elements for prolonged periods of time. 

Testosterone and cortisol represent hormonal parameters that provide a snapshot of the current anabolic status of an athlete [11]. Traditionally, these two endocrine biomarkers are utilized in male athletes to identity and prevent overtraining [51]. No significant differences in testosterone, cortisol, or T:C ratio were observed between groups in this study. Nevertheless, a trend was observed for decreased cortisol concentrations in the probiotic group. This is in agreement with previous work which found no effect of probiotics on cortisol concentrations during a period of intense military training [49]. However, one study in a non-athletic population reported lower cortisol responses in participants which received a prebiotic (soluble fiber compounds which enhance the growth of gut microbiota) supplement daily for three weeks [52]. Thus, there is precedent for gut modulatory substances producing a reduced cortisol response in humans. Furthermore, coupled with the probiotic attenuation of TNF-α in our study, coinciding lower average cortisol levels in the probiotic group may indicate a better homeostatic balance for health, recovery, and physiological adaptations.

Zonulin is a protein which plays a central role in modulating intercellular tight junctions in the intestinal endothelium [53]. Of late, this protein has been proposed as a novel circulating marker of intestinal permeability [54]. In the present study, we found no differences in plasma zonulin concentrations following our 12-week intervention. Previous work found that 14 weeks of probiotic supplementation resulted in significantly decreased levels of fecal zonulin, indicating an improvement in intestinal barrier integrity [16]. While previous literature have observed changes in circulating zonulin following probiotic interventions, it is possible that fecal measurements of zonulin may have been a more sensitive marker to detect changes in our healthy participants. Some investigations have observed compromised gut permeability in response to an acute exercise stress in trained participants following endurance and interval training [41,55]. Thus, future work should seek to characterize intestinal permeability following acute resistance exercise or a competitive event. Finally, it is possible that the strain utilized in our investigations may have no measureable effect on circulating zonulin as a proxy marker for gut impairment. 

It has been proposed that probiotic supplementation may improve gastrointestinal function resulting in increased absorption of dietary protein [26] which may contribute to enhanced adaptations over the course of a training intervention. In a mouse model, 6 weeks of *Lactobacillus plantarum* produced augmented strength, muscle mass, and type I muscle fiber number, while improving endurance swimming performance [56]. Also, in a computer-controlled in vitro model of the small intestine, *Bacillus coagulans* GBI-30, 6086 enhanced amino acid absorption while improving colon health [26]. As such, we postulated that in addition to possible effects on immune function, that probiotic supplementation may increase the benefits of post-workout nutrition. In the current study, we observed no differences in any measure of strength or physical performance between groups. However, baseline differences in strength measures (i.e., squat and deadlift), and the lack of training volume analysis limited the interpretation of these data. Additionally, we found no preferential effects of probiotic supplementation on muscle thickness and body composition. To date, only two manuscripts have investigated the effect of probiotic administration on resistance training adaptations. The first investigation [29] found no ergogenic benefit of a probiotic supplement on muscular strength and power following 12 weeks of circuit-resistance training which is in concert with previous work in endurance athletes reporting no effect of probiotics on performance [17,19,20,57]. The second study found no benefit of daily *Bacillus subtilis* (five billion CFU) supplementation on measures of physical performance following 10 weeks of offseason training in female Division I volleyball and soccer athletes [27]. However, Toohey et al., [27] did observe significant improvements in body composition when probiotic was administered with a protein carbohydrate beverage which were not supported in this study with male participants. While we utilized the same probiotic strain as the previous study in female athletes (*Bacillus subtilis* DE111), we provided our athletes with a smaller daily dose (one billion CFU) of probiotic. Additionally, the females enrolled in the previous investigation [27] who experienced significant improvements had an average body fat percentage of 25.1%, whereas our male participants began the intervention with an average body fat of 14.3% and much less room for improvement. Therefore, the apparent discrepancy between our results and existing data to date could result from subtle sex and dosage-dependent differences, in addition to dissimilarities in baseline body composition measures. 

## 5. Conclusions

Our data indicate that 12 weeks of probiotic supplementation provides no beneficial effect regarding body composition, physical performance, hormonal status, or gut permeability while attenuating circulating TNF-α concentrations in college athletes following offseason training. College athletes typically undergo periods of elevated stress both physically and mentally, which may negatively affect recovery and adaptation. While decreased TNF-α levels as a result of probiotic supplementation may be promising, no other performance or body composition benefits were found which leaves the practical relevance of decrease inflammation in athletes convoluted. One limitation of our study is that we did not collect any direct measures of stressors (e.g., questionnaires) beyond biochemical markers and detailing the training regimen. This includes additional assessments that were not assessed in this study regarding sleep patterns, perception of academic stress, social influences, and quantification of training volume and load, which would provide a better picture of the demands of college student-athletes, and potential practical relevance of probiotic administration. Furthermore, it appears that our study was statistically under-powered to detect modest effects in some biomarkers (e.g., cortisol). As the current study was limited by the size of the athletic team studied (25 athletes), additional work is need in trained athletes with a larger sample size. Moreover, as the effects of probiotics may be dose, strain, and sex dependent, further research is needed.

## Figures and Tables

**Table 1:** Twelve-week offseason resistance training program.

**Phase 1-Eccentric**	**Weeks 1–4**				
**Day 1**	**Sets × Reps**	**Day 2**	**Sets × Reps**	**Day 3**	**Sets × Reps**
Squat	4 × 8−5 w/:03-:05s ECC	Dead Lift	4 × 8−5	Hang Clean	4 × 8−5
Box Jump	4 × 4	Single Hops	4 × :08s	Single Leg Box Jumps	4 × 5
Mobility	3 × 10	Single Leg Box Squats	4 × 5	Inverted Row	4 × 10
Bench Press	4 × 8−5 w/:03-:05s ECC	Scap Angels	3 × 10	Single Arm Dumbbell Bench	4 × 8−6
3 Point Row	4 × 8 w/:03-:05s ECC	Dumbbell Incline Press	4 × 8−5	Exercise Ball Core	4 × 6
GHD Falls	3 × 8	Banded Swimmers Row	4 × 10	Sled Push	4 × 1
		Banded Face Pull	4 × 10	Banded Hip Flexor Pull	4 × 10
		6 Pack Scaps YTA	3 × 6 :03s ECC	-	-
**Circuit 1** 50s work:10s rest × 3	**Circuit 2**	**Circuit 1**	**Circuit 2**	**Circuit 1**	**Circuit 2**
Int/Ext Shoulder Rotation	Split Squat	Airex Floor Touches	Keiser Resisted Lunge	Band Pull-Aparts	Box Step-ups
Plank	TGU	Banded Hip Lifts	Banded X Walks	Keiser SL Twist	Ab Wheel
HK Chops	Pullup	Shoulder Raises	Side Plank Row	Kettle Bell Lunge	Med Ball Slams
**Phase 2-Isometric**	**Weeks 5–8**				
**Day 1**	**Sets × Reps**	**Day 2**	**Sets × Reps**		
Hang Clean	4 × 6−4	Dead Lifts	4 × 6−4		
Mobility	3 × 5	SL Hexagon Hops	4 × :08s		
Squat	4 × 6−4 w/:03-:05s ISO	W/Y Negatives	3 × 8		
Lateral Box Jump	4 × 4	SL Pistol Squat	4 × 5		
DB Incline Bench Press	4 × 6−4 w/:03s ISO	Bench Press	4 × 6−4 w/:03s ISO		
Bear Row	4 × 8−6 w/:03s ISO	Battle Rope Variations	3 × :30s		
Sled Push	3 × 1	Black Burns	3 × 5		
Lateral Lunge	3 × 8	SL RDL Reaches	3 × 8		
Farmers Carry	3 × 3	TRX Archor Row	3 × 8		
Pull-ups	2 × 8, 1 × 6 w/:03s ISO	Landmine Rotation and Press	3 × 8		
Standing Keiser Twists	3 × 10	Med Ball Fielding Drill	3 × 10		
		Exercise Ball Knee Drives	3 × 10		
**Phase 3-Concentric**	**Weeks 9–12**				
**Day 1**	**Sets × Reps**	**Day 2**	**Sets × Reps**	**Day 3**	**Sets × Reps**
Squat	4 × 4−2	Dead Lift	4 × 4−2	Hang Clean	4 × 4−2
Box Jump	4 × 4	lateral Bound	4 × 6	Dead Bugs	4 × 5
Mobility	3 × 5	Inverted Row	3 × 8	Cross-Over ATYT	3 × 15
Incline Bench	4 × 4−3	Bench Press	4 × 4−3	Mobility	3 × 10
3 Point Row	4 × 5−3	Med Ball Chest Pass	4 × 5	Single Arm Bench	4 × 4−3
Hip Lift	4 × 6	BlackBurns	4 × 5	6 Pack Scaps	4 × 6
Battle Rope Variations	3 × :30s	Single Leg Squat	4 × 5	Lateral Sled Pull	3 × 1
Inline Board Lunge	3 × 8	Side Plank Row	3 × 8	Keiser Single Arm Single Leg Row	3 × 8
Pull-up	3 × 8	Band Pull-Aparts	3 × 10	Med Ball Slams	3 × 10
Keiser Low Row	3 × 8	Valslide Lateral Lunge	3 × 8	Towel Pull-ups	3 × 8
Supine Bridge w/Cross Body Med Ball Throw	3 × 10	Landmine Touches	3 × 10	Vertimax Pull Over	3 × 10
		Prone Hip Openers	3 × 10		

ECC = Eccentric Emphasis; ISO = Isometric Emphasis; Int/ext = Interior and Exterior; GHD = Glute Hamstring Developer; HK = Half-kneeling; SL = Single Leg; TGU = Turkish Get-Up; RDL = Romanian Deadlift; DB = Dumbbell; Cross-over ATYT = Arms form each letter in a cross-over band set-up. W/Y Negatives = Arms form each letter in a cross-over band set-up.

**Table 2:** Strength, performance, and body composition changes following 12weeks of offseason training.

Variable	Treatment	PRE	Covariate	POST	F	*p*	ƞ^2^	95% Confidence Interval	
								Lower	Upper
Squat 1RM (kg)	PRO	116.8 ± 17.1	124.9	141.8 ± 11.2	0.459	0.505	0.020	139.2	159.4
	PL	133.0 ± 32.0		162.2 ± 40.0				143.6	164.7
Deadlift 1RM (kg)	PRO	139.9 ± 12.2	151.3	169.4 ± 21.0	0.375	0.547	0.019	172.2	188.9
	PL	162.8 ± 40.5		188.0 ± 39.1				168.7	185.2
Standing Long Jump (m)	PRO	2.46 ± 0.17	2.50	2.55 ± 0.21	0.046	0.833	0.003	2.53	2.64
	PL	2.54 ± 0.28		2.64 ± 0.19				2.54	2.66
Pro-Agility (sec)	PRO	4.62 ± 0.17	4.60	4.49 ± 0.22	1.152	0.300	0.071	4.41	4.55
	PL	4.58 ± 0.20		4.50 ± 0.23				4.46	4.60
10yd Sprint (sec)	PRO	1.99 ± 0.86	1.86	1.69 ± 0.12	0.852	0.371	0.054	1.63	1.77
	PL	1.70 ± 0.11		1.66 ± 0.09				1.57	1.73
Body Fat (%)	PRO	14.7 ± 5.6	14.3	14.9 ± 4.8	2.119	0.161	0.096	13.7	15.7
	PL	14.0 ± 4.9		13.4 ± 4.8				12.9	14.6
RF Muscle Thickness (cm)	PRO	2.39 ± 0.44	2.44	2.51 ± 0.47	0.166	0.687	0.008	2.49	2.64
	PL	2.50 ± 0.28		2.60 ± 0.29				2.46	2.62
VL Muscle Thickness (cm)	PRO	1.73 ± 0.23	1.79	1.78 ± 0.23	0.513	0.481	0.023	1.81	1.89
	PL	1.86 ± 0.33		1.93 ± 0.33				1.83	1.91

Data presented as mean ± SD.

**Table 3:** Changes in Biochemical Markers Following 12 weeks of Offseason Training.

Variable	Treatment	PRE	Covariate	POST	F	*p*	ƞ^2^	95% Confidence Interval	
								Lower	Upper
TNF-α (pg/mL)	PRO	2.32 ± 0.93	2.37	2.07 ± 0.76	5.86	0.024 *	0.210	1.69	2.49
	PL	2.42 ± 1.49		2.78 ± 0.95				2.35	3.18
LN IL-10 (pg/mL)	PRO	2.79 ± 0.97	2.95	2.89 ± 1.08	0.032	0.860	0.001	2.89	3.22
	PL	3.12 ± 0.88		3.27 ± 1.02				2.91	3.25
Zonulin (ng/mL)	PRO	10.6 ± 2.11	10.14	10.8 ± 2.23	0.010	0.921	<0.001	9.68	11.04
	PL	9.67 ± 4.32		9.86 ± 4.27				9.60	11.02
Testosterone (nmol/L)	PRO	15.3 ± 6.59	15.7	15.8 ± 6.50	1.89	0.183	0.79	14.8	17.4
	PL	16.2 ± 4.56		17.8 ± 4.46				16.0	18.8
Cortisol (nmol/L)	PRO	656.3 ± 237.7	662.8	579.4 ± 183.2	3.41	0.078	0.134	488.9	678.0
	PL	669.9 ± 224.1		709.5 ± 247.4				606.6	803.5
T/C Ratio	PRO	0.024 ± 0.009	0.025	0.030 ± 0.013	0.464	0.503	0.021	0.024	0.036
	PL	0.025 ± 0.008		0.027 ± 0.009				0.020	0.033
Total WBC (×10^9^/L)	PRO	5.97 ± 1.50	5.84	7.08 ± 1.85	0.235	0.632	0.011	5.95	8.21
	PL	5.71 ± 1.31		7.46 ± 2.00				6.28	8.64
SIgA Secretion Rate (μg/min)	PRO	105.2 ± 56.4	123.1	176.6 ± 86.5	1.59	0.222	0.070	138.6	236.7
	PL	141.1 ± 97.2		156.1 ± 98.3				96.0	194.1
LN SIgM Secretion Rate (μg/min)	PRO	8.11 ± 1.45	8.07	8.84 ± 1.07	0.452	0.509	0.021	8.32	9.30
	PL	8.02 ± 1.40		8.55 ± 1.50				8.10	9.07

Data presented as mean ± SD. LN = natural log transformation. * significantly different from PL.

## References

[1] Petersen A.M.W., Pedersen B.K. (2005). The anti-inflammatory effect of exercise. J. Appl. Physiol..

[2] Gleeson M., McDonald W., Cripps A., Pyne D., Clancy R., Fricker P. (1995). The effect on immunity of long-term intensive training in elite swimmers. Clin. Exp. Immunol..

[3] Mackinnon L.T., Ginn E., Seymour G.J. (1993). Decreased salivary immunoglobulin A secretion rate after intense interval exercise in elite kayakers. Eur. J. Appl. Physiol. Occup. Physiol..

[4] Mackinnon L.T., Jenkins D.G. (1993). Decreased salivary immunoglobulins after intense interval exercise before and after training. Med. Sci. Sports Exerc..

[5] Fahlman M.M., Engels H.-J. (2005). Mucosal IgA and URTI in American college football players: A year longitudinal study. Med. Sci. Sports Exerc..

[6] Gepner Y., Hoffman J.R., Shemesh E., Stout J.R., Church D.D., Varanoske A.N., Zelicha H., Shelef I., Chen Y., Frankel H. (2017). Combined effect of Bacillus coagulans GBI-30, 6086 and HMB supplementation on muscle integrity and cytokine response during intense military training. J. Appl. Physiol..

[7] Hoffman J.R., Gepner Y., Stout J.R., Hoffman M.W., Ben-Dov D., Funk S., Daimont I., Jajtner A.R., Townsend J.R., Church D.D. (2016). β-Hydroxy-β-methylbutyrate attenuates cytokine response during sustained military training. Nutr. Res..

[8] Main L.C., Dawson B., Heel K., Grove J.R., Landers G.J., Goodman C. (2010). Relationship between inflammatory cytokines and self-report measures of training overload. Res. Sports Med..

[9] Lang C.H., Frost R.A., Nairn A.C., MacLean D.A., Vary T.C. (2002). TNF-α impairs heart and skeletal muscle protein synthesis by altering translation initiation. Am. J. Physiol.-Endocrinol. Metab..

[10] Mann J.B., Bryant K.R., Johnstone B., Ivey P.A., Sayers S.P. (2016). Effect of physical and academic stress on illness and injury in division 1 college football players. J. Strength Cond. Res..

[11] Lee E.C., Fragala M.S., Kavouras S.A., Queen R.M., Pryor J.L., Casa D.J. (2017). Biomarkers in sports and exercise: Tracking health, performance, and recovery in athletes. J. Strength Cond. Res..

[12] Carfagno D.G., Hendrix J.C. (2014). Overtraining syndrome in the athlete: Current clinical practice. Curr. Sports Med. Rep..

[13] Pyne D.B., West N.P., Cox A.J., Cripps A.W. (2015). Probiotics supplementation for athletes—Clinical and physiological effects. Eur. J. Sport Sci..

[14] Borchers A.T., Selmi C., Meyers F.J., Keen C.L., Gershwin M.E. (2009). Probiotics and immunity. J. Gastroenterol..

[15] Lescheid D.W. (2014). Probiotics as regulators of inflammation: A review. Funct. Foods Health Dis..

[16] Lamprecht M., Bogner S., Schippinger G., Steinbauer K., Fankhauser F., Hallstroem S., Schuetz B., Greilberger J.F. (2012). Probiotic supplementation affects markers of intestinal barrier, oxidation, and inflammation in trained men; a randomized, double-blinded, placebo-controlled trial. J. Int. Soc. Sports Nutr..

[17] Michalickova D., Minic R., Dikic N., Andjelkovic M., Kostic-Vucicevic M., Stojmenovic T., Nikolic I., Djordjevic B. (2016). *Lactobacillus helveticus* Lafti L10 supplementation reduces respiratory infection duration in a cohort of elite athletes: A randomized, double-blind, placebo-controlled trial. Appl. Physiol. Nutr. Metab..

[18] Gleeson M., Bishop N.C., Oliveira M., Tauler P. (2011). Daily probiotic’s (*Lactobacillus casei* Shirota) reduction of infection incidence in athletes. Int. J. Sport Nutr. Exerc. Metab..

[19] Cox A.J., Pyne D.B., Saunders P.U., Fricker P.A. (2010). Oral administration of the probiotic Lactobacillus fermentum VRI-003 and mucosal immunity in endurance athletes. Br. J. Sports Med..

[20] West N.P., Pyne D.B., Cripps A.W., Hopkins W.G., Eskesen D.C., Jairath A., Christophersen C.T., Conlon M.A., Fricker P.A. (2011). Lactobacillus fermentum (PCC^®^) supplementation and gastrointestinal and respiratory-tract illness symptoms: A randomised control trial in athletes. Nutr. J..

[21] Coqueiro A.Y., de Oliveira Garcia A.B., Rogero M.M., Tirapegui J. (2017). Probiotic supplementation in sports and physical exercise: Does it present any ergogenic effect?. Nutr. Health.

[22] Gleeson M., Bishop N.C., Oliveira M., McCauley T., Tauler P., Lawrence C. (2012). Effects of a *Lactobacillus salivarius* probiotic intervention on infection, cold symptom duration and severity, and mucosal immunity in endurance athletes. Int. J. Sport Nutr. Exerc. Metab..

[23] Kekkonen R.A., Vasankari T.J., Vuorimaa T., Haahtela T., Julkunen I., Korpela R. (2007). The effect of probiotics on respiratory infections and gastrointestinal symptoms during training in marathon runners. Int. J. Sport Nutr. Exerc. Metab..

[24] Shing C.M., Peake J.M., Lim C.L., Briskey D., Walsh N.P., Fortes M.B., Ahuja K.D., Vitetta L. (2014). Effects of probiotics supplementation on gastrointestinal permeability, inflammation and exercise performance in the heat. Eur. J. Appl. Physiol..

[25] Jäger R., Purpura M., Stone J.D., Turner S.M., Anzalone A.J., Eimerbrink M.J., Pane M., Amoruso A., Rowlands D.S., Oliver J.M. (2016). Probiotic *Streptococcus thermophilus* FP4 and *Bifidobacterium breve* BR03 supplementation attenuates performance and range-of-motion decrements following muscle damaging exercise. Nutrients.

[26] Keller D., Van Dinter R., Cash H., Farmer S., Venema K. (2017). *Bacillus coagulans* GBI-30, 6086 increases plant protein digestion in a dynamic, computer-controlled in vitro model of the small intestine (TIM-1). Benef. Microbes.

[27] Toohey J.C., Townsend J.R., Johnson S.B., Toy A.M., Vantrease W.C., Bender D., Crimi C.C., Stowers K.L., Ruiz M.D., VanDusseldorp T.A. (2018). Effects of Probiotic (*Bacillus subtilis*) Supplementation during Offseason Resistance Training in Female Division I Athletes. J. Strength Cond. Res..

[28] Georges J., Lowery R.P., Yaman G., Kerio C., Ormes J., McCleary S.A., Sharp M., Shields K., Rauch J., Silva J. (2014). The effects of probiotic supplementation on lean body mass, strength, and power, and health indicators in resistance trained males: A pilot study. J. Int. Soc. Sports Nutr..

[29] Ibrahim N.S., Muhamad A.S., Ooi F.K., Meor-Osman J., Chen C.K. (2018). The effects of combined probiotic ingestion and circuit training on muscular strength and power and cytokine responses in young males. Appl. Physiol. Nutr. Metab..

[30] McCrory M.A., Molé P.A., Gomez T.D., Dewey K.G., Bernauer E.M. (1998). Body composition by air-displacement plethysmography by using predicted and measured thoracic gas volumes. J. Appl. Physiol..

[31] Anderson L.J., Erceg D.N., Schroeder E.T. (2015). Utility of multi-frequency bioelectrical impedance compared to deuterium dilution for assessment of total body water. Nutr. Diet..

[32] Siri W.E. (1956). The gross composition of the body. Adv. Biol. Med. Phys..

[33] Jajtner A.R., Hoffman J.R., Scanlon T.C., Wells A.J., Townsend J.R., Beyer K.S., Mangine G.T., McCormack W.P., Bohner J.D., Fragala M.S. (2013). Performance and muscle architecture comparisons between starters and nonstarters in National Collegiate Athletic Association Division I women’s soccer. J. Strength Cond. Res..

[34] Abe T., Fukashiro S., Harada Y., Kawamoto K. (2001). Relationship between sprint performance and muscle fascicle length in female sprinters. J. Physiol. Anthropol. Appl. Hum. Sci..

[35] Hoffman J. (2006). Norms for Fitness, Performance, and Health.

[36] Chicharro J.L., Lucía A., Pérez M., Vaquero A.F., Ureña R. (1998). Saliva composition and exercise. Sports Med..

[37] Schipper R.G., Silletti E., Vingerhoeds M.H. (2007). Saliva as research material: Biochemical, physicochemical and practical aspects. Arch. Oral Biol..

[38] Dietz C., Peterson B. (2012). Triphasic Training: A Systematic Approach to Elite Speed and Explosive Strength Performance.

[39] Green S., Salkind N., Akey T. (2000). Methods for controlling type I error across multiple hypothesis tests. Using SPSS for Windows: Analysing and Understanding Data.

[40] Van Wijck K., Lenaerts K., Van Loon L.J., Peters W.H., Buurman W.A., Dejong C.H. (2011). Exercise-induced splanchnic hypoperfusion results in gut dysfunction in healthy men. PLoS ONE.

[41] Pugh J.N., Impey S.G., Doran D.A., Fleming S.C., Morton J.P., Close G.L. (2017). Acute high-intensity interval running increases markers of gastrointestinal damage and permeability but not gastrointestinal symptoms. Appl. Physiol. Nutr. Metab..

[42] Martarelli D., Verdenelli M.C., Scuri S., Cocchioni M., Silvi S., Cecchini C., Pompei P. (2011). Effect of a probiotic intake on oxidant and antioxidant parameters in plasma of athletes during intense exercise training. Curr. Microbiol..

[43] Hirose L., Nosaka K., Newton M., Laveder A., Kano M., Peake J., Suzuki K. (2004). Changes in inflammatory mediators following eccentric exercise of the elbow flexors. Exerc. Immunol. Rev..

[44] Peake J., Nosaka K.K., Suzuki K. (2005). Characterization of Inflammatory Responses to Eccentric Exercise in Humans. Exerc. Immunol. Rev..

[45] Hardin B.J., Campbell K.S., Smith J.D., Arbogast S., Smith J., Moylan J.S., Reid M.B. (2008). TNF-α acts via TNFR1 and muscle-derived oxidants to depress myofibrillar force in murine skeletal muscle. J. Appl. Physiol..

[46] Trochimiak T., Hübner-Woźniak E. (2012). Effect of exercise on the level of immunoglobulin A in saliva. Biol. Sport.

[47] Mackinnon L.T., Chick T.W., Van As A., Tomasi T.B. (1989). Decreased secretory immunoglobulins following intense endurance exercise. Res. Sports Med. Int. J..

[48] Steerenberg P.A., Asperen I.A., Amerongen A.N., Biewenga J., Mol D., Medema G. (1997). Salivary levels of immunoglobulin A in triathletes. Eur. J. Oral Sci..

[49] Tiollier E., Chennaoui M., Gomez-Merino D., Drogou C., Filaire E., Guezennec C.Y. (2007). Effect of a probiotics supplementation on respiratory infections and immune and hormonal parameters during intense military training. Mil. Med..

[50] Clancy R., Gleeson M., Cox A., Callister R., Dorrington M., D’este C., Pang G., Pyne D., Fricker P., Henriksson A. (2006). Reversal in fatigued athletes of a defect in interferon γ secretion after administration of *Lactobacillus acidophilus*. Br. J. Sports Med..

[51] Hayes L.D., Grace F.M., Baker J.S., Sculthorpe N. (2015). Exercise-induced responses in salivary testosterone, cortisol, and their ratios in men: A meta-analysis. Sports Med..

[52] Schmidt K., Cowen P.J., Harmer C.J., Tzortzis G., Errington S., Burnet P.W. (2015). Prebiotic intake reduces the waking cortisol response and alters emotional bias in healthy volunteers. Psychopharmacology.

[53] Fasano A. (2012). Intestinal permeability and its regulation by zonulin: Diagnostic and therapeutic implications. Clin. Gastroenterol. Hepatol..

[54] Moreno-Navarrete J.M., Sabater M., Ortega F., Ricart W., Fernandez-Real J.M. (2012). Circulating zonulin, a marker of intestinal permeability, is increased in association with obesity-associated insulin resistance. PLoS ONE.

[55] Mach N., Fuster-Botella D. (2017). Endurance exercise and gut microbiota: A review. J. Sport Health Sci..

[56] Chen Y.-M., Wei L., Chiu Y.-S., Hsu Y.-J., Tsai T.-Y., Wang M.-F., Huang C.-C. (2016). *Lactobacillus plantarum* TWK10 supplementation improves exercise performance and increases muscle mass in mice. Nutrients.

[57] Gill S.K., Teixeira A.M., Rosado F., Cox M., Costa R.J.S. (2016). High-dose probiotic supplementation containing *Lactobacillus casei* for 7 days does not enhance salivary antimicrobial protein responses to exertional heat stress compared with placebo. Int. J. Sport Nut. Exerc. Metab..

